# Challenges to overcome obstacles for pediatric donor heart availability

**DOI:** 10.1016/j.jhlto.2025.100395

**Published:** 2025-09-24

**Authors:** Norihide Fukushima

**Affiliations:** Senri Kinran University, 5–25-1 Fujishiro-dai, Suita, Osaka, 565-0873, Japan

**Keywords:** Pediatric heart transplantation, Xenotransplantation, Donor evaluation and management, Donation after circulatory death (DCD)

## Abstract

Although surgical techniques for congenital heart disease (CHD), including hypoplastic left heart syndrome, have progressively advanced, pediatric heart transplantation remains the most effective surgical option for complex CHD and cardiomyopathy with severe heart failure. However, donor heart availability for children continues to be limited. This article reviews past and current challenges in overcoming obstacles to pediatric donor heart availability.

## Background

Christiaan Barnard performed the first clinical heart transplantation (HTx) in Cape Town, South Africa, on December 3, 1967.^1^ Although the recipient died of pneumonia 18 days after HTx, the operation opened the door for the Stanford group to further develop the procedure in humans. advance clinical HTx. With respect to infants, Adrian Kantrowitz in New York attempted HTx in a newborn only three days later.^2^ However, neonatal transplantation was not attempted again until 1984, 16 years later.^3^, ^4^

Before the definition of brain death in children under 5 years of age was approved in 1987,^5^ various other sources—such as other animal species,^3^ human fetuses,^2^ and dying human infants—were proposed. Even after the official definition was established and neonatal and infant organ donation gained wider acceptance globally, the shortage of suitable donors remained critical. Consequently, numerous efforts to increase donor availability have continued to the present day.

### Challenges in overcoming legal, social, and ethical obstacles

In September 1968, an ad hoc committee of Harvard Medical School issued a report on the “Definition of Brain Death.”^6^ The committee concluded that life support could be withdrawn from patients diagnosed with brain death and that, with appropriate consent, their hearts could be procured for transplantation. Following this, the number of adult HTx procedures steadily increased. However, at that time, the criteria for brain death specifically excluded “young children.” The Presidential Commission’s 1981 report^7^ continued to define brain death specially excluded children under 5 years of age, reflecting the prevailing assumption that the immature brain was more resistant to insults leading to death.^8^, ^9^ Consequently, alternative sources of donor hearts—including other animal species, human fetuses, and dying human infants—were proposed.

### The use of hearts from anencephalic infants

Adrian Kantrowitz in New York attempted HTx in a 19-day-old newborn with Ebstein anomaly, who had previously undergone a Blalock–Taussig shunt, using the heart of an anencephalic neonate, just three days after Barnard’s first HTx.^2^ The recipient died 6.5 hours after the procedure, and Kantrowitz did not pursue further clinical transplantation. On December 6, 1968, Denton Cooley in Texas performed a heart–lung transplantation in a 2-month-old infant with severe pulmonary hypertension due to a congenital atrioventricular canal defect, also using the heart of an anencephalic neonate; the infant died from pulmonary insufficiency 14 hours postoperatively.^10^ The use of organs from anencephalic newborns has been reported worldwide for more than 25 years, primarily in abdominal organ transplantation.^11^ However, anencephalic infants do not meet established criteria for brain death, raising significant ethical and legal concerns.^12^, ^13^ Most anencephalic infants are stillborn, and those born alive typicaly die within one week without extraordinary interventions. Other organ systems beyond the brain may also be abnormal. Furthermore, the incidence of anencephaly has declined, partly due to prenatal screening and elective termination. After the publication of the 1986 guidelines for the determination of brain death in children - which allowed organ donation from brain-dead children under 5 years of age,^5^ pediatric HTx using anencephalic donors decreased and was eventually abandoned.

### Xenogeneic HTx (Xeno-HTx)

For nearly a decade after the first HTx, clinical transplantation was limited to only a handful of pioneering institutions, and virtually no group pursued research in neonatal HTx except Leonard L. Bailey’s team at Loma Linda University. His laboratory conducted experiments with orthotopic allo-HTx in newborn goats. Using cyclosporine-A immunosuppression alone, they achieved remarkable survival, maturation, and even reproductive capacity.^14^ Recipients of cross-species grafts from lamb to goat also demonstrated unprecedented survival of up to 47 days.^15^ Building on these results, the team began to explore the use of infant baboons as donors for neonates with hypoplastic left heart syndrome (HLHS), establishing a colony of infant baboons. They investigated baboon-derived infections and human–baboon immunologic responses through specialized HLA typing, two-way mixed lymphocyte cultures, and compatibility testing with human neonates using an ex vivo perfusion model.^16^

On October 26, 1984, a neonate with HLHS—known as “Baby Fae”—underwent xeno-HTx with a carefully selected infant baboon donor.^3^ She survived for 20 days, but despite thorough evaluation, the precise cause of death remains uncertain.^17^ Nevertheless, her case heightened awareness worldwide and directly paved the way for the first successful neonatal HTx, performed again as treatment for HLHS, in November 1985. That patient is now a 40-year-old man living and working in Las Vegas. Baby Fae’s legacy endures in the hundreds of neonates and infants worldwide who are alive today as a result of pediatric HTx.

However, the shortage of infant donors remained severe, and Bailey’s group focused on survival studies of orthotopic xeno-HTx using juvenile baboon recipients in both concordant and discordant models. Xenograft bridging to allo-HTx was evaluated in a series of five juvenile baboons, suggesting that a clinical protocol for xeno-to-allo bridging might be feasible.^18^ Two consecutive series of orthotopic xeno-HTx from rhesus monkey donors demonstrated long-term survival of up to 502 days.^19^

As the pig is anatomically and physiologically similar with human and its gestation period is short and they rapidly grow, the pig seems to be the most suitable species for the clinical application of xenotransplantation. Therefore, Investigations of discordant (pig-to-baboon) transplantation focused on adsorption of naturally occurring xenoreactive antibodies at the time of grafting.^20^ This strategy, combined with pre-transplant total lymphoid irradiation and both pre- and post-transplant immunosuppression, successfully prevented hyperacute rejection and achieved survival of up to 24 days.

Nevertheless, xeno-HTx entails far greater uncertainties than conventional therapies. Multiple advisory bodies on xenotransplantation, including the British Paediatric Association^21^ and the Medical Research Council, have recommended that therapeutic research should not involve children if it could be conducted equally well in adults. Large-scale and high-risk xenotransplantation trials in children could only be ethically justified once major uncertainties had been resolved through adult studies. Accordingly, both the FDA and WHO have advised that initial xenotransplantation trials should be performed in adults rather than children. Thus, although Bailey’s group and others attempted to continue animal experiments in preparation for clinical pediatric xeno-HTx into the mid-2000s, they ultimately abandoned these efforts.

Although it took nearly 30 years, advances in genetic engineering—particularly CRISPR/Cas9 technology^22^—have made it possible to modify pig organs to improve their immunological and physiological compatibility. Preclinical studies have shown that the survival of primate recipients of life-saving xenografts can now be extended beyond 2 years.^23^

In the first 2 patients who received genetically modified pig hearts, histological analysis revealed minimal immune cell infiltration but indicated capillary endothelial injury with interstitial edema and early fibrosis.^22^ To further advance xenotransplantation, strategies to overcome antibody-mediated rejection (AMR) are essential. Several areas of research still require progress: (i) identifying the optimal genetic engineering profile for donor pigs; (ii) defining the most effective immunosuppressive protocol; and (iii) establishing sensitive microbiological tests to prevent and promptly detect latent zoonotic infections.

## Challenge to overcome immunological obstacles

### ABO incompatible HTx

Generally, donor and recipient blood types must be matched. However, in young infants, the immune system is immature, and the production of anti-A and anti-B antibodies remains insufficient until approximately 12 to 14 months of age. This immunologic immaturity permits ABO-incompatible (ABOi) HTx, thereby expanding donor options. Since the first ABOi infant HTx in Canada in 1996,^24^ multiple studies have demonstrated no significant differences in outcomes or rejection-free survival between ABOi and ABO-compatible (ABOc) infant HTx.^25^, ^26^

A first multicenter study published in 2013,^27^ involving 57 patients who received 58 ABOi transplants, demonstrated that antibodies against the donor blood group were significantly lower post-transplant than pre-transplant in ABOi recipients. Freedom from death or retransplantation was 100%, 96%, and 69% at 1, 5, and 10 years, respectively, comparable to outcomes in ABOc transplant patients of the same age receiving similar immunosuppressive regimens. The Pediatric Heart Transplant Society reported that 85 of 502 infant transplants (17%) were ABOi, confirming equivalent 1-year survival and freedom from rejection compared with ABOc recipients, despite most centers reserving ABOi transplants for sicker patients.^28^

Given these comparable outcomes, an increasing number of countries have adopted ABOi HTx as a strategy to address donor shortages. According to United Network for Organ Sharing (UNOS) policy, acceptable anti-A or anti-B antibody titers are <1:16.^29^ In the United Kingdom, Irvings et al reported 12 patients—five of whom were older than 2 years—with titers ≥1:16 who underwent HTx. Four patients experienced early AMR within 15 days, and three subsequently died, although not necessarily due to high antibody titers. In contrast, survival among patients with titers <1:16 was 89%.^30^ These findings suggest that while ABOi HTx can expand the recipient pool, careful consideration of antibody levels remains essential, and its broader application requires further investigation.

### Positive crossmatch

Prior to listing for HTx, most centers evaluate each recipient for antibodies against non-self　human leukocyte antigens (HLAs) using a panel reactive antibody (PRA) test. If preformed antibodies to potential donors are detected, those donors are avoided to prevent hyperacute or acute AMR early after HTx. Recipients with a high PRA percentage (commonly defined as >10%) are considered sensitized, which poses a challenge as it can exclude multiple potential donor options. Consequently, sensitized children face longer waitlist durations and higher waitlist mortality.^31^, ^32^

Some centers may still proceed with transplantation in the presence of known donor-specific antibodies, a practice known as positive crossmatch (XM) transplantation. A review of the UNOS registry demonstrated that elevated PRA levels were associated with worse post-transplant mortality overall.^33^ A prospective, multi-institutional observational cohort study from the Clinical Trials in Organ Transplantation in Children program reported that sensitized recipients, as defined by their criteria, had reduced freedom from acute AMR and cellular rejection. However, freedom from death, re-transplantation, or rejection with hemodynamic compromise at 12 months was comparable between sensitized and non-sensitized patients.^34^ In Japan, prospective complement-dependent cytotoxicity crossmatching has been applied in all pediatric HTx since 2000. Notably, no cases of hyperacute rejection or early death within the first 6 months post-HTx were observed, and 10-year survival was 92.9%.^35^

Recently, flow cytometry XM for detecting anti-HLA antibodies has been recommended for recipient selection worldwide. However, flow cytometry is often considered overly sensitive, as not all donor-specific antibodies (DSAs) identified are cytotoxic to the cardiac graft. Within the classical complement pathway, alloantibodies bind to HLA antigens on donor graft cells and subsequently recruit C1q, which is essential for the formation of the membrane attack complex and cell lysis.^36^ Therefore, the detection of circulating complement-binding DSAs may be more clinically relevant.^37^, ^38^ At Stanford University, an assay for detecting C1q-binding antibodies was developed and applied in pediatric recipients. This approach identified a subset of patients at increased risk for AMR early after transplantation.^39^ Although C1q or C3d-binding DSA assays are not definitive methods for identifying DSAs that cause AMR, they may serve as valuable adjuncts in guiding immunotherapy.

Although desensitization represents a recipient-focused rather than donor-focused strategy, several trials have explored its role. Holt et al reported perioperative desensitization with plasmapheresis, thymoglobulin, and cyclophosphamide in 17 patients with PRA >10%, 13 of whom also had a positive complement-dependent cytotoxicity XM. This cohort demonstrated survival outcomes comparable to those reported in the International Society for Heart and Lung Transplantation (ISHLT) registry.^40^ However, most patients experienced early rejection, with many developing recurrent or hemodynamically significant episodes within the first 6 months post-HTx.^40^ Subsequently, The Hospital for Sick Children reported intra- and postoperative plasma exchange, thymoglobulin induction, and post-transplant plasmapheresis in 12 children with positive XMs. In this cohort, 3-month and 1-year survival rates were 89% and 71%, respectively. Nine patients developed AMR and seven developed Grade 2R acute cellular rejection, while one patient died from rejection-related hemodynamic compromise. Consistent with prior studies, no cases of AMR occurred beyond 6 months post-HTx.^41^

An alternative approach that addresses these challenges is virtual crossmatching (virtual XM). Virtual XM involves comparing the recipient’s known anti-HLA antibodies against the donor’s HLA profile. Although some centers have established criteria for proceeding with transplants despite a positive virtual XM, a retrospective positive XM is associated with significantly worse 2-year survival after pediatric HTx.^42^ Steven Zangwill et al^43^ reported that virtual XM was 100% sensitive but only 72% specific for predicting a negative T-cell XM and 86% specific for predicting a negative B-cell XM. Notably, the false negatives were only weakly positive, suggesting that many donor hearts flagged by virtual XM would likely have been clinically acceptable.

## Donation after circulatory death (DCD)

### Original DCD

Although not widely recognized among the general public or even many physicians, the first HTx performed by Christiaan N. Barnard on December 3, 1967, was carried out using a DCD donor.^1^ A 54-year-old man with end-stage ischemic heart disease received the heart of a motor vehicle accident victim who had sustained severe brain injury. The donor’s ventilator was withdrawn, and her heart ceased beating naturally due to hypoxia within 10-12 minutes. Death was certified following 5 minutes of absent electrocardiographic activity, spontaneous respiration, and reflexes. The donor was then placed on cardiopulmonary bypass (CPB), and the heart was resuscitated. The graft was perfused using a CPB machine and transplanted with the Lower–Shumway technique. The recipient subsequently died of *Pseudomonas* pneumonia after 18 days.

On January 6, 1968, Shumway, Stinson, and colleagues performed the first HTx from a controlled DCD donor. After a diagnosis of brain death by neurologists and declaration of death, the donor was extubated, and the heart was procured. Prior to the publication of the first formal brain death criteria by the Ad Hoc Committee of the Harvard Medical School in July 1968,^6^ many HTxs were performed using DCD donors. Due to the shortage of brain-dead donors, several groups investigated DCD HTx; however, outcomes were generally poor. As a result, animal experiments with DCD donors gradually declined, while the use of brain-dead donors increased thereafter.

In the late 1980s, Shirakura et al^44^, ^45^ and Gundry et al^46^ independently reported that administration of steroids, prostaglandins, and calcium channel blockers could induce cardiac arrest without ventricular fibrillation following asphyxia, and the heart was harvested 30 minutes after electric cardiac arrest to confirm the animal’s death and was transplanted orthotopically. All hearts regained sinus rhythm spontaneously, without the need for cardioversion, and all animals were successfully weaned from CPB without inotropic support. In Gundry’s experiment, recipient baboons survived until donor hearts were rejected, up to 34 days after HTx.^46^

Between 2004 and 2006, three orthotopic infant HTx from DCD donors were performed at Denver Children’s Hospital in recipients with a mean age of 2.2 months.^47^ The mean time to cardiac arrest after withdrawal of life support was 18.3 ± 8.3 minutes. All three recipients survived beyond 6 months. Nevertheless, the adoption of pediatric DCD HTx has been limited, largely due to ethical concerns as well as logistical and operational challenges.

An analysis of UNOS data demonstrated that only seven pediatric DCD heart transplants were performed between 2004 and 2022, with only one center performing more than two procedures.^48^ Similarly, an analysis of the ISHLT Registry reported 23 pediatric DCD heart transplants worldwide by 2018.^49^ Thus, despite acceptable survival rates, the use of pediatric DCD donors for HTx remains extremely limited globally.

### Logistics for expanding HTx

Recently, two approaches have enabled successful transplantation using DCD hearts. The first involves ex vivo organ machine perfusion (OMP) to resuscitate donor hearts following a period of circulatory arrest and cardiac death. The second, normothermic regional perfusion (NRP), was first adopted and reported by a UK group.

#### Ex vivo OMP

Although not yet approved by the Food and Drug Administration for pediatric use, OMP offers several advantages in pediatric HTx. Cathlyn et al. reported their single-center experience using the TransMedics Organ Care System (OCS) for ex vivo perfusion in pediatric HTx.^50^ Donors weighing ≥40 kg were selected to ensure compatibility with the OCS device, enabling the use of larger donor hearts. Post-transplant, all patients demonstrated normal left ventricular function at discharge. Over a median follow-up of 11.9 months, there were no deaths. These findings suggest that ex vivo perfusion is a valuable technique for adolescent HTx.

#### NRP

NRP establishes in situ reperfusion of the heart and other organs with oxygenated blood using extracorporeal membrane oxygenation or CPB. Following NRP procurement, the heart can be transported either via cold storage or using the OCS. NRP offers several significant advantages: (1) reduction of donor warm ischemic time, (2) correction of metabolic abnormalities associated with circulatory death, and (3) a controlled procurement process that facilitates safe dissection for all teams involved.

Historically, in the 1990s, the present author conducted animal experiments on multi-organ transplantation from DCD donors using percutaneous CPB.^40^ All transplanted hearts resumed spontaneous beating, and all animals were successfully weaned from CPB without inotropic support.

Recent use of NRP for the heart was first adopted and reported by a UK group. This approach was adapted from a method previously used successfully for abdominal organ procurement. The UK group has reported experiences with both OMP and NRP for DCD HTx, although the NRP experience has been limited due to technical constraints in the number of hospitals able to perform it. Nevertheless, their most recent five-year report suggests that outcomes with NRP-procured hearts may surpass those achieved with OMP.^51^

In the United States, James et al reported their early experience with 18 DCD HTx procedures using thoraco-abdominal NRP via central CPB at 37 °C through median sternotomy.^52^ Hearts were harvested from adult donors using standard donation methods and preserved immersely. All hearts were deemed suitable for transplantation and were successfully implanted. Careful intraoperative management of DCD donors, aimed at optimizing metabolic conditions, may improve graft function in adult and adolescent heart transplant recipients.

#### Pediatric donor management to optimize donor heart utilization

The donor myocardium may sustain varying degrees of injury from multiple causes, including autonomic storm secondary to brain death, cardiac arrest, thoracic trauma, and cardiopulmonary resuscitation maneuvers.^53^ In particular, cardiac dysfunction resulting from an autonomic storm after brain death is often referred to as *neurogenic stress cardiomyopathy* in heart donors.^54^ With appropriate donor management, left ventricular dysfunction in an otherwise healthy donor is often transient, and post-transplant outcomes using such recovered donor hearts are acceptable in both pediatric and adult recipients.53., 55, 56, 57, 58

Hearts from donors with a history of cardiac arrest and cardiopulmonary resuscitation may also be suitable for transplantation, provided that cardiac function has recovered and there is no evidence of significant underlying disease or ischemic electrocardiographic changes.53., 55 According to the 2020 ISHLT registry report, recipients of donors who died from anoxia or head trauma had the highest 1-year survival (89.9%), whereas the lowest 1-year survival (84.1%) was observed in recipients of donors who died from cerebrovascular accident or stroke.^59^

### Serial echocardiography evaluation

Echocardiography can be used to evaluate myocardial and valvular function, myocardial hypertrophy, and the presence of congenital malformations. Although global or even regional ventricular dysfunction may occur following brain death, these wall motion abnormalities are often reversible within hours.^53^ Therefore, serial echocardiography should be performed before discarding a donor heart due to myocardial dysfunction.53., 57

Even if hemodynamics and cardiac systolic function appear to be preserved, the myocardium may still be considered compromised if high doses of inotropes are required to maintain stability. Thus, assessment of left ventricular function should be carried out after reducing inotrope dosages as much as possible. Because patients with brain injury are usually maintained in a relatively dehydrated state to prevent cerebral edema, the heart may appear to contract normally due to reduced preload. For accurate assessment of systolic function, central venous pressure at the time of evaluation should be maintained at 8-10 mmHg. It is also essential to correct hemoglobin concentration, electrolyte balance, and acid–base status during this evaluation.

Furthermore, proper evaluation of donor cardiac function requires management of diabetes insipidus, adjustment of peripheral vascular tone, and restoration of β-adrenergic receptor (BAR) affinity for adrenaline within the myocardium. This can be achieved by administering antidiuretic hormone (ADH) via a central venous line while optimizing circulating blood volume.

### Continuous administration of ADH

High serum adrenaline concentrations, as well as the administration of high intravenous doses of adrenaline, are significantly associated with a reduction in myocardial BAR density.^60^, ^61^ Therefore, the use of adrenaline should be minimized whenever possible. With respect to catecholamine dosing, a donor heart requiring more than 15 μg/kg/min of dopamine is generally considered an extended-criteria donor, particularly in the presence of abnormal ECG or echocardiographic findings. In most cases, dopamine requirements below 15 μg/kg/min are considered acceptable.

Low-dose arginine vasopressin not only treats diabetes insipidus but also reduces inotropic requirements and has been associated with preserved kidney, liver, and cardiac graft function.^56^ Because vasopressin also improves vascular tone and enhances BAR responsiveness, it should be administered even in patients with low urine output. In fact, vasopressin can improve hemodynamics and renal function, thereby increasing urine output, as has been demonstrated in patients following cardiotomy or with septic shock.

Vasopressin should be administered via a central venous line at a continuous infusion rate of 0.01-0.02 U/kg/h (or 0.5-1 U/h) following an initial bolus dose of 0.5-1 U. Once hemodynamic stability is achieved, norepinephrine—and subsequently adrenaline—can be tapered rapidly in favor of dopamine or dobutamine. When both circulating and exogenous adrenaline levels return to the normal range, the heart rate typically stabilizes between 90 and 120 beats per minute. Vasopressin infusion should be continued until all organs are cannulated and systemic heparinization has been administered to maintain stable hemodynamics during procurement.^53^

Diabetes insipidus may cause high urine output, hypernatremia, hypokalemia, increased serum osmolality, reduced circulating blood volume, and intracellular dehydration, which can lead to liver or renal dysfunction and arrhythmias. To prevent these complications, serum sodium should be maintained at 135-150 mEq/L, potassium at 3.8-4.5 mEq/L, hematocrit above 30%, blood glucose between 120 and 180 mg/dL, and core body temperature between 35.5 °C-36.5 °C.

### Donor evaluation and management system in Japan

Following the initial determination of brain death in a potential organ donor, specialized transplant management physicians—often former cardiac transplant surgeons and currently transplant cardiologists—are dispatched to the procurement hospital.^53^ These medical consultants assess donor heart function and determine transplant suitability. They also initiate intensive donor management, including administration of ADH as described above, minimization of intravenous inotropes, and optimization of organ function prior to the arrival of the procurement team.

In Japan, the Japan Organ Transplant Network (JOT) contacts heart transplant centers only after a comprehensive evaluation of donor heart function has been completed. This practice reduces the refusal rate of initially compromised donor hearts. The heart procurement team physician subsequently reassesses donor heart function by echocardiography to confirm suitability for the intended recipient. Between April 2011 and April 2025, 92 of 100 consecutive pediatric donor hearts referred to JOT were successfully transplanted. Notably, no early deaths within the first 6 months post-transplant were observed, and the 10-year survival rate was 92.9%.^35^

## Financial support

None.

## Declaration of Competing Interest

The authors declare that they have no known competing financial interests or personal relationships that could have appeared to influence the work reported in this paper.
